# Long-term (6 and 12 months) follow-up of two prospective, randomized, controlled phase III trials of photodynamic therapy with BF-200 ALA and methyl aminolaevulinate for the treatment of actinic keratosis

**DOI:** 10.1111/bjd.12158

**Published:** 2013-04

**Authors:** T Dirschka, P Radny, R Dominicus, H Mensing, H Brüning, L Jenne, L Karl, M Sebastian, C Oster-Schmidt, W Klövekorn, U Reinhold, M Tanner, D Gröne, M Deichmann, M Simon, F Hübinger, G Hofbauer, G Krähn-Senftleben, F Borrosch, K Reich, C Berking, P Wolf, P Lehmann, M Moers-Carpi, H Hönigsmann, K Wernicke-Panten, S Hahn, G Pabst, D Voss, M Foguet, B Schmitz, H Lübbert, R-M Szeimies

**Affiliations:** 1 Private Dermatological Practice CentreWuppertal, Germany; 2 Faculty of Health, University Witten-HerdeckeWitten, Germany; 3 Private Dermatological PracticeFriedrichshafen, Germany; 4 Private PracticeDülmen, Germany; 5 Dermatological AmbulatoryHamburg, Germany; 6 Day Hospital for Allergy and Skin DiseasesKiel, Germany; 7 Private PracticeSellspeicher, Kiel, Germany; 8 Dermatological PracticeSoest, Germany; 9 Private PracticeMahlow, Germany; 10 Private Practice for Skin and Genital Diseases AltenkirchenAltenkirchen, Germany; 11 Private Dermatological PracticeGilching, Germany; 12 Medical Centre BonnBonn, Germany; 13 Dermatosurgery Center NördlingenNördlingen, Germany; 14 Dermatological PracticeBerlin, Germany; 15 Dermatological PracticeGelnhausen, Germany; 16 Dermatological Practice, Clinical Study CentreBerlin, Germany; 17 Private PracticeWuppertal, Germany; 18 University Hospital Zurich, Clinic of DermatologyZurich, Switzerland; 19 Cutis Sana DermatologistsBlaubeuren, Germany; 20 Centre for Dermatology VechtaVechta, Germany; 21 SCIderm GmbHHamburg, Germany; 22 Clinic for Dermatology und Allergology, Ludwig-Maximilian-University MunichMunich, Germany; 23 Department of Dermatology and Venereology, Research Unit for Photodermatology, Medical University of GrazGraz, Austria; 24 HELIOS Hospital WuppertalWuppertal, Germany; 25 Private Practice for Skin Diseases, Operative and Aesthetic DermatologyMunich, Germany; 26 University Hospital for DermatologyVienna, Austria; 27 Accovion GmbHEschborn, Germany; 28 Focus Clinical Drug Development GmbHNeuss, Germany; 29 Biofrontera Bioscience GmbHLeverkusen, Germany; 30 Klinikum Vest, Department of Dermatology and AllergologyRecklinghausen, Germany

## Abstract

**Background:**

Two phase III trials of photodynamic therapy (PDT) with BF-200 ALA, a recently approved nanoemulsion formulation of 5-aminolaevulinic acid (ALA) demonstrated high clearance rates in mild-to-moderate actinic keratosis (AK). The comparison to a registered methyl aminolaevulinate (MAL) cream demonstrated significantly superior total patient clearance rates.

**Objectives:**

To evaluate long-term efficacy and safety of PDT for AK 6 and 12 months after the last PDT with BF-200 ALA, MAL or placebo.

**Methods:**

The follow-up phase (FUP) was performed with patients of two phase III studies. Both studies compared BF-200 ALA with placebo, one of the studies additionally with MAL. Overall recurrence rates and various subgroups (light source, lesion severity, lesion location, complete responders after first PDT) were assessed 6 and 12 months after the last PDT.

**Results:**

Recurrence rates were similar for BF-200 ALA and MAL, with a tendency to lower recurrence rates for BF-200 ALA. The proportion of patients who were fully cleared during PDT and remained completely clear for at least 12 months after PDT were 47% for BF-200 ALA (both studies) and 36% for MAL treatment. The subgroup that was illuminated with narrow wavelength LED lamps reached 69% and 53% for BF-200 ALA (both studies, respectively) and 41% for MAL. No safety concerns were reported.

**Conclusions:**

The FUP data confirmed the high efficacy and safety of PDT with BF-200 ALA. The slightly lower recurrence rates after BF-200 ALA treatment compared with MAL treatment enhanced the better treatment outcome due to the significantly superior efficacy.

Actinic keratosis (AK) is defined as squamous cell carcinoma (SCC) *in situ*.^1^,^2^ It represents the most common neoplasia affecting fair-skinned subjects in sun-exposed body areas such as the face and scalp. AKs may progress to SCC, therefore necessitating treatment.^3^,^4^ As photodynamic therapy (PDT) with 5-aminolaevulinic acid (ALA), or its derivative methyl aminolaevulinate (MAL), combines high efficacy with an excellent cosmetic outcome, it is recommended as one of the first-line treatments in this indication.^1^

Formulations containing ALA have frequently been used clinically, but are often restrained by their short-term stability and poor skin penetration. BF-200 ALA is a new nanoemulsion-based gel formulation containing 7·8% ALA (10% ALA hydrochloride), which overcomes these drawbacks, displaying an improved stability of ALA in the aqueous formulation and an enhanced penetration into the epidermis.^5^ Based on these advantages, lower ALA concentrations are sufficient for an excellent therapeutic outcome, which was recently demonstrated in two phase III studies of the treatment of AK, one of them in comparison to a registered MAL formulation.^6^,^7^

Here we present the recurrence rates of subjects treated with BF-200 ALA or MAL PDT in two phase III trials and the percentage of patients who were totally cleared of AK 12 months after PDT. Safety issues such as new lesions and skin cancer in the treatment area are also reported.

## Material and methods

Data were collected during the follow-up period (FUP) of two randomized, placebo-controlled, multicentre phase III clinical studies.^6^,^7^ All subjects in both studies entered the FUP 3 months after the last treatment and were invited to two FUP visits, the first at 6 months and the second at 12 months after the last PDT. Patient assessments at the FUP visits were analysed distinct from the study and summarized in a separate report. The studies were approved by the responsible ethics committees and the competent authorities (BfArM, Germany; Swissmedic, Switzerland; BASG, Austria) and performed according to the national drug laws, the guidelines of Good Clinical Practice and the Declaration of Helsinki.

During the clinical parts of the studies, eligible subjects with four to eight mild-to-moderate AK lesions (Olsen grade I or II^8^) on the face or scalp were treated with BF-200 ALA containing ALA 78 mg g^−1^ (now registered as Ameluz®; Biofrontera, Leverkusen, Germany), or a registered MAL cream (Metvix®; Galderma, Düsseldorf, Germany) containing 160 mg g^−1^ of MAL, or placebo matching the BF-200 ALA formulation. After roughening of lesion surfaces and cleaning the skin with alcohol, medications were applied for 3 h. Then, remnant gel or cream was carefully wiped off and lesions were illuminated with red light either with narrow-spectrum lamps (LED lamps: Aktilite® CL 128, Photocure, Oslo, Norway; Omnilux PDT™, Photo Therapeutics Inc., Montgomeryville, PA, U.S.A.) or broad-spectrum light sources (PhotoDyn® 750/505; Hydrosun Medizintechnik GmbH, Müllheim, Germany; Waldmann® PDT 1200 L; Waldman Medizintechnik, Villingen-Schwenningen, Germany) as described in detail in the study protocols.^6^,^7^ Treatment efficacy and cosmetic outcome were assessed 3 months after PDT. In cases of remaining lesions, a second treatment was performed at this time point. The final assessment was then carried out after 3 more months.

### Study population

All subjects who completed the clinical trials and did not withdraw their informed consent were enrolled in the FUP to assess long-term treatment effects with respect to recurrences of AK lesions and safety issues. All subjects with complete remission of AK lesions in the treatment areas were included in the subject-based recurrence analysis. Further, the design of the FUP of study ALA-AK-CT003 (superiority study over placebo^6^), in addition to following completely cleared patients, allowed the follow-up of every individual lesion that was completely cleared after the last PDT. This lesion-based FUP analysis was not performed in study ALA-AK-CT002 (comparative study with the registered MAL cream)^7^ as only lesions of complete responders were considered in this trial.

### Study plan

**Fig. 1:**
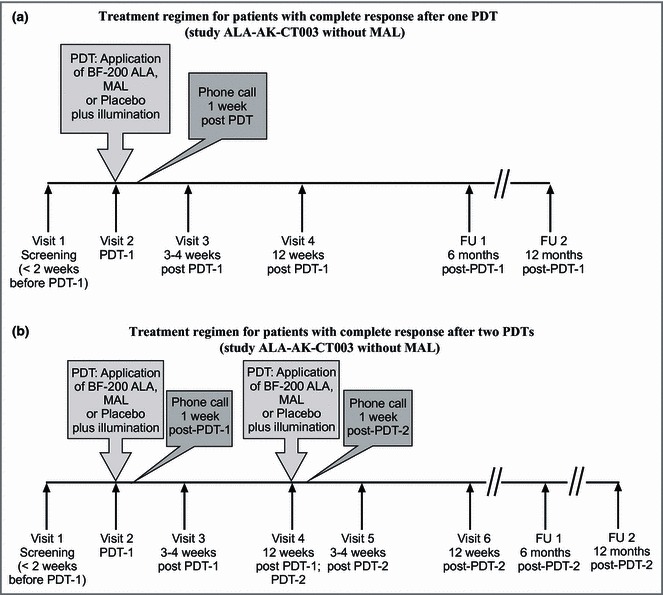
Treatment regimen of the clinical phases and follow-up (FU) phases of studies ALA-AK-CT002 and ALA-AK-CT003. Assessment of photodynamic therapy (PDT) efficacy was scheduled 3 months after PDT. Subsequently, completely cleared patients entered FUP with two visits 6 and 12 months after PDT (a). In case of residual lesions 3 months after the first PDT, a second PDT was performed (b) with a final assessment of efficacy 3 months later. MAL, methyl aminolaevulinate.

To analyse patient and lesion long-term efficacy, patient visits to the study centres were scheduled 6 and 12 months after the last PDT (Fig. 1). If recurrent lesions occurred, patients received an additional AK therapy chosen by the investigator. In addition to recurrent lesions, new AK lesions, skin cancers and cosmetic outcome in the treatment areas were recorded at the scheduled visits. Serious adverse events (SAEs) were recorded for both studies.

### Recurrence rate assessment

Patients who were completely cleared at the end of the clinical study, i.e. 3 months after the last PDT, were included in the analysis. Patients or lesions completely cleared 3 months after the last PDT, but then diagnosed and/or treated at or before FUP visit 1 (FUP1; i.e. 6 months after the last PDT) or FUP visit 2 (FUP2; i.e. 12 months after the last PDT) were assessed as recurrent. In study ALA-AK-CT003, all totally cleared lesions were examined individually, both in patients with all lesions cleared and in those with lesions remaining. The following of each individual lesion was not done in study ALA-AK-CT002 (comparative study), where only complete responders were considered during FUP. The consequence of this is that if a subject in study ALA-AK-CT002 received any AK therapy between the end of the clinical phase and FUP2, or if a subject was flagged as recurrent, but the number of recurrent AKs was not recorded, all of the subjects’ lesions were defined as recurrent. This leads to an overestimation of lesion-based recurrence rates in study ALA-AK-CT002.

If a patient was recurrent at FUP1 they were also counted as recurrent at FUP2, irrespective of an AK therapy that may have cleared the patient in the meantime.

Nonrecurrence rates were evaluated using the life-table method for grouped data. Two values were calculated: (i) the probability that a patient or lesion, completely cleared at the end of the clinical phase of the study, remains completely clear up to the 6- and 12-month FUP visits (*P*_i_ for visit i, i = 1, 2); and (ii) the probability that a patient or lesion is fully cleared during PDT and remains completely clear until the follow-up visit at 6 or 12 months after the last PDT (*P*_i_ × CR_P_ or *P*_i_ × CR_L_; see below).

### Biometric analysis

Data were obtained independently for both studies and reported for the intent-to-treat (ITT; ALA-AK-CT002) or the full-analysis set (FAS; ALA-AK-CT003) population, respectively. Patient-based and lesion-based recurrence rates were estimated at both follow-up visits, i.e. after 6 months (FUP1) and after 12 months (FUP2), based on an approach of time-to-event analysis. As only two time points were considered, the life-table method for grouped data was chosen. This is the grouped data equivalent to the Kaplan–Meier estimator. To take withdrawals or patients lost to FUP adequately into account it is assumed that the withdrawal times are continuously uniformly distributed on the time interval between the visits. This allows censoring half of the dropouts at the beginning of the interval and the other half at the end of the interval. Table 1 shows how the estimators for subject-based recurrence rates have been calculated.

**Table 1:** Calculation of life-table estimates for subject-based recurrence rates

Visit name	Visit (i)	CR_Pi_ = CR_P(i−1)_ − R_i_ − L_i_	R_i_	L_i_	*f*_i_ = 1 − R_i_/(CR_Pi_ − L_i_/2)	*P*_i_ = *P*_(i−1)_ × *f*_i_
3 months after last PDT	0	CR_P0_	0	0	1	1
Follow-up after 6 months	1	CR_P1_	R_1_	L_1_	*f*_1_	*P*_1_
Follow-up after 12 months	2	CR_P2_	R_2_	L_2_	*f*_2_	*P*_2_

CR_P0_, number of subjects totally cleared of lesions at end-of-study visit 3 months after the last photodynamic therapy (PDT); CR_Pi_, number of subjects with complete remission of all actinic keratosis lesions in the target area(s) at current visit; R_i_, number of subjects with at least one recurrent lesion in target area(s) between preceding and current visit; L_i_, number of subjects lost to follow-up or withdrawn between preceding and current visit; *f*_i_, probability of remaining clear for subjects with complete remission at preceding visit; *P*_i_, probability of remaining clear up to current visit.

Recurrence rates were assessed for treatment groups and various subgroups (e.g. by centre, sex, age, skin type, target area, light spectrum and AK severity at baseline if appropriate). The approaches were performed taking into consideration: (i) the number of subjects or lesions completely cleared at the end-of-study visit 3 months after the last PDT; (ii) the number of subjects/lesions with complete remission in the target area(s) at the current visit; (iii) the number of subjects with at least one recurrent lesion or new recurrent lesions in the target area(s) between the preceding and the current visit; and (iv) the number of subjects/lesions lost to FUP or withdrawn between the preceding and the current visit.

For the FUP, probabilities (*P*_i_) for subjects remaining fully clear up to the respective visit (visit i, i = 1, 2) were assessed according to life-table estimates as shown in Table 1 (probabilities regarding totally cleared lesions were performed similarly).

Firstly, the number of subjects with complete clearance of all AK lesions in the target area(s) at the current visit (CR_Pi_) was calculated by subtracting subjects with at least one recurrent lesion in the target area(s) between the preceding and the current visit (R_i_), and the number of subjects lost to FUP or withdrawn between the preceding and the current visit (L_i_) from the totally cleared patient group at the last visit (CR_Pi−1_):


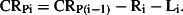


Secondly, for subjects with complete remission at the preceding visit, the probability of remaining cleared could then be calculated:


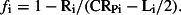


Finally, the probability of remaining clear up to the current visit could be estimated using the formula:


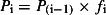


Furthermore, the probability that a patient or lesion, respectively, is cleared during treatment and remains clear up to the current visit was estimated by multiplying the *P*_i_ of the assessed treatment group and the corresponding patient-based (CR_P0_) or lesion-based clearance rate (CR_L0_) 3 months after the last PDT (end of clinical trial); this term is defined as *P*_i_ * CR_P_ or *P*_i_ * CR_L,_ respectively:


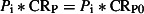


Example for *P*_i_ * CR_P_ calculation

The *P*_1_ value of 0·827 achieved at the FUP1 for BF-200 ALA-treated patients in the ALA-AK-CT003 study indicates that 82·7% of the patients who were completely cleared 3 months after the last PDT (66·3%) remain completely clear at this time point. In addition, the corresponding product *P*_1_ × CR_P_ (0·827 × 0·663) of 0·548 takes the respective efficacy 3 months after the last PDT into consideration and reflects the probability of a patient entering PDT to be cleared at FUP1 (for values see Tables 2 and 4).

**Table 2:** Overview of demographics

	ALA-AK-CT003		ALA-AK-CT002		
		
	BF-200 ALA	Placebo	BF-200 ALA	MAL	Placebo
*Clinical trial*					
Patients enrolled in clinical study, *n*	122		600		
Patients randomized, *n*	81	41	248	247	76
FAS/ITT population, *n* (%)	80 (100)	40 (100)	248 (100)	246 (100)	76 (100)
Premature discontinuation, *n*	4	4	7	7	8
Complete responders 3 months after last PDT, *n* (CR_P_, %)	53 (66·3)	5 (12·5)	194 (78·2)	158 (64·2)	13 (17·1)
After 1st PDT	38 (47·5)	4 (10·0)	120 (48·4)	91 (37·0)	3 (3·9)
After 2nd PDT	15 (38·5)	1 (3·0)	74 (57·8)	67 (43·2)	10 (13·7)
Totally cleared lesions 3 months after last PDT, *n* (CR_L_, %)	369 (81·1)	46 (20·9)	1359 (90·4)	1295 (83·2)	182 (37·1)
*Follow-up phase (FUP)*					
Patients entering FUP (FAS/ITT), *n* (%)	77 (96·3)	37 (92·5)	241 (97·2)	240 (97·6)	68 (89·5)
FUP1	72 (93·5)	34 (91·9)	238 (98·8)	236 (98·3)	66 (97·1)
FUP2	71 (92·2)	32 (86·5)	236 (97·9)	232 (96·7)	64 (94·1)
Patients completing FUP, *n* (%)	70 (90·9)	32 (86·5)	233 (94·0)	231 (93·9)	64 (94·1)
Lost to FUP, *n*	7	5	8	9	4
Complete responders entering FUP^a^, *n* (%)	53 (66·3)	5 (12·5)	192 (77·4)	157 (63·8)	13 (17·1)
FUP1	51 (63·8)	4 (10·0)	188 (75·8)	154 (62·6)	13 (17·1)
FUP2	49 (61·3)	4 (10·0)	185 (74·6)	154 (62·6)	13 (17·1)
Lesions considered for recurrence rate in FUP, *n* (%)^b^	353	45	1147	958	84

ALA, 5-aminolaevulinic acid; FAS, full analysis set; FUP1/2, follow-up visits 1 and 2; ITT, intent-to-treat; MAL, methyl aminolaevulinate; PDT, photodynamic therapy.

a Only subjects who were completely clear 3 months after the last PDT were considered for evaluation of recurrence rates;

b only lesions of complete responders were considered in all patient groups of study ALA-AK-CT002. If in study ALA-AK-CT002 patients were treated for actinic keratosis between FUP visits or the number of recurrent lesions was not noted, all lesions of the patient were counted as recurrent.

**Table 4:** Patient-based life table: number of subjects still cleared at 6-month (FUP1) and 12-month (FUP2) follow-up visits in study ALA-AK-CT003

	BF-200 ALA (*n* = 53)			Placebo (*n* = 5)		
		
	Subjects completely cleared^a^, %	*P*_i_	*P*_i_ * CR_P_	Subjects completely cleared^a^, %	*P*_i_	*P*_i_ * CR_P_
FUP1	82·4	0·827	0·548	50·0	0·556	0·069
FUP2	69·4	0·706	0·468	50·0	0·556	0·069

AK, actinic keratosis; ALA, 5-aminolaevulinic acid; *P*_i,_ probability that a completely cleared patient remains completely clear up to FUP1 or FUP2; *P*_i_ * CR_P_, probability that a patient is fully cleared from all lesions during photodynamic therapy and remains totally clear until the follow-up visit (product of *P*_i_ and efficacy rate at the end of the clinical trial).

a The percentage is calculated according to the number of patients at the respective visit.

For the calculation of *P*_i_ * CR data for comparator compounds, the required data were obtained from publications of controlled, randomized phase III trials.^9^–^12^ The following values were taken from those references and entered in the calculation: number of completely cleared patients at the end of the study, patients entering FUP, patients lost to FUP (wherever data are provided, otherwise set to 0), and number of patients with at least one recurrent lesion during FUP. The calculation was as described above and illustrated in Table 1.

New lesions or skin cancers observed in the target area were assessed by descriptive statistics.

## Results

### Patients

A total of 663 patients from both phase III studies entered the FUP, accounting for 93·4% (114 patients) and 96·1% (549 patients) of the randomized subjects of studies ALA-AK-CT003 and ALA-AK-CT002, respectively. Of these, 63·3% (420 patients) were complete responders. Six hundred and thirty subjects completed the follow-up (102 subjects in study ALA-AK-CT003, 528 subjects in study ALA-AK-CT002) while 33 patients discontinued prematurely (Table 2).

**Fig. 2:**
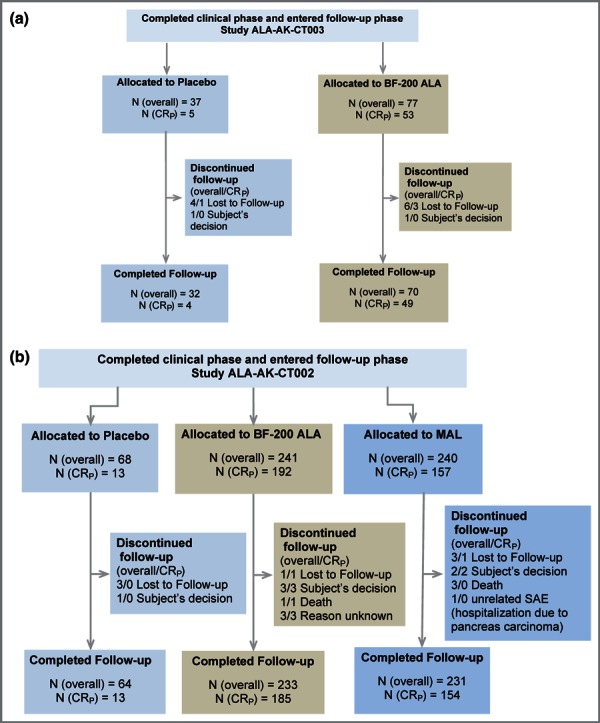
Allocation of patients in the follow-up phases (FUPs) of studies ALA-AK-CT003 (a) and ALA-AK-CT002 (b). The numbers of patients entering and completing FUP are indicated along with the cause of discontinuation during the FUP. CR_P_, complete responders, i.e. patients without remaining lesions 3 months after the last treatment with photodynamic therapy; MAL, methyl aminolaevulinate; SAE, serious adverse event.

A flow chart of the patient disposition is presented in Figure 2. Patient characteristics are summarized in Table 3. Results are shown for the ITT/FAS follow-up population.

**Table 3:** Summary of patient characteristics entering follow-up phase (intent-to-treat/full analysis set)

	ALA-AK-CT003		ALA-AK-CT002		
		
	BF-200 ALA (*n* = 77)	Placebo (*n* = 37)	BF-200 ALA (*n* = 241)	MAL (*n* = 240)	Placebo (*n* = 68)
Sex, *n* (%)					
Male	69 (89·6)	29 (78·4)	208 (86·3)	199 (82·9)	54 (79·4)
Female	8 (10·4)	8 (21·6)	33 (13·7)	41 (17·1)	14 (20·6)
Age (years)					
Mean ± SD	70·4 ± 5·2	71·3 ± 6·4	70·1 ± 7·2	71·0 ± 7·0	71·7 ± 6·8
Range	58–82	60–85	39–87	44–85	51–84
Severity of AK at baseline, *n*					
Mild (grade I)	15	2	33	37	3
Moderate (grade II)^a^	38	3	159	120^b^	10
Target area, *n*					
A (face and forehead)	31	2	120	107	4
B (bald scalp)	13	3	47	23	5
A + B	9	0	25	27	4
Light spectrum, *n*					
Narrow	27	2	104	84	5
Broad	26	3	88	73	8

AK, actinic keratosis; ALA, 5-aminolaevulinic acid; MAL, methyl aminolaevulinate.

a Patients with at least one AK assessed as grade II at start of clinical trial;

b one severe (grade III) lesion included.

### Efficacy

#### Patient nonrecurrence rates

Rates of nonrecurrent patients in the FUP of both pivotal studies are summarized in Table 4 (for study ALA-AK-CT003) and Table 5 (for study ALA-AK-CT002). No significant differences became apparent between BF-200 ALA- and MAL-treated subjects at FUP1. More than 80% of the patients were still complete responders 6 months after the last PDT with BF-200 ALA (in both studies) or MAL.

**Table 5:** Patient-based life table: number of subjects still cleared at 6-month (FUP1) and 12-month (FUP2) follow-up visits in study ALA-AK-CT002

	BF-200 ALA (*n* = 192)			MAL (*n* = 157)			Placebo (*n* = 13)		
			
	Subjects completely cleared^a^, %	*P*_i_	*P*_i_ * CR_P_	Subjects completely cleared^a^, %	*P*_i_	*P*_i_ * CR_P_	Subjects completely cleared^a^, %	*P*_i_	*P*_i_ * CR_P_
FUP1	80·9	0·812	0·647	81·8	0·821	0·537	84·6	0·846	0·162
FUP2	58·4	0·592	0·472	55·2	0·555	0·363	76·9	0·769	0·147

AK, actinic keratosis; ALA, 5-aminolaevulinic acid; MAL, methyl aminolaevulinate; *P*_i,_ probability that a completely cleared patient remains completely clear up to FUP1 or FUP2; *P*_i_ * CR_P_, probability that a patient is fully cleared from all lesions during photodynamic therapy and remains totally clear until the follow-up visit (product of *P*_i_ and efficacy rate at the end of the clinical trial).

a The percentage is calculated according to the number of patients at the respective visit.

A slightly larger variation was observed at FUP2 after 12 months, when for BF-200 ALA patients, 58·4% in study ALA-AK-CT002 and 69·4% in ALA-AK-CT003 were still nonrecurrent. For MAL, 55·2% of the patients were still completely cleared. The difference at FUP2 between the patient clearance rates in the BF-200 ALA groups of the two pivotal trials may be partly due to the larger percentage of patients with moderate lesions (Olsen grade II) in study ALA-AK-CT002 (Table 3). In both studies at FUP2, recurrence rates for Olsen grade II patients treated with BF-200 ALA were about 14% points higher than for patients with only mild (Olsen grade I) lesions (compare Table 7).

In study ALA-AK-CT002, the probability (*P*_2_) in the BF-200 ALA group of remaining cleared 12 months after the last PDT was 0·592, and that for the MAL group 0·555. In study CT003, the probability of remaining completely cleared after BF-200 ALA treatment at 12 months FUP was 0·706 (Tables 4 and 5).

Combining efficacy and recurrence rates, the probability of being totally clear 12 months after the last treatment (*P*_2_ * CR_P0_) was 0·472 and 0·468 (ALA-AK-CT002 and ALA-AK-CT003, respectively) for BF-200 ALA-treated patients, and 0·363 for MAL-treated patients (ALA-AK-CT002; Tables 4 and 5). In spite of the lower recurrence rate in study ALA-AK-CT003, the overall *P*_2_ * CR_P_ for BF-200 ALA were very similar in studies ALA-AK-CT003 and ALA-AK-CT002. This apparent discrepancy is due to the higher proportion of patients treated with broad-spectrum lamps in study ALA-AK-CT003, causing a lower overall efficacy (78·2% vs. 66·3% for CT002 and CT003, respectively; see Table 2).

Because only fully cleared patients were considered for FUP, this generates a very small group of patients on placebo. In addition, patients in the placebo group presenting with all lesions totally cleared may represent a specific selection of patients with a strong ability for AK clearance. Thus, recurrence rates in the placebo groups are difficult to interpret.

#### Lesion recurrence rate

A total of 1359 (90·4%) and 369 (81·1%) BF-200 ALA-treated lesions were totally cleared at the end of the clinical parts of studies ALA-AK-CT002 and ALA-AK-CT003, respectively, and 1295 (83·2%) of MAL-treated lesions in study ALA-AK-CT002 (Table 2). For the placebo groups, 182 (37·1%) and 46 (20·9%) of the lesions were cleared 12 weeks after the last treatment in the respective studies. Of the BF-200 ALA-treated lesions 1147 and 353 from the two studies entered the follow-up phase, and 958 of the MAL-treated lesions. Eighty-four and 45 lesions were monitored in the respective placebo groups (Table 2). In contrast to study ALA-AK-CT003 in which every individual lesion was monitored during FUP, in study ALA-AK-CT002 only lesions of complete responders entered the FUP, reducing the total number of completely cleared lesions analysed. This again leads to a small number of lesions followed up in the placebo group, rendering any interpretation of the results in this group questionable.

In the ALA-AK-CT002 study, the overall lesion recurrence rates after 6 months were 7·0% for BF-200 ALA-treated patients and 6·6% for MAL-treated patients; 3·6% were recurrent in the placebo group. Lesion recurrence in ALA patients at FUP1 in ALA-AK-CT003 was 7·4%, and 6·1% for patients in the placebo group. After 12 months, adding recurrence rates of FUP1 and FUP2, BF-200 ALA patients in study ALA-AK-CT002 had a lesion recurrence rate of 21·7%, MAL patients of 25·4% and placebo patients of 15%. In study ALA-AK-CT003, the combined lesion recurrence rates after 12 months were 16·7% for BF-200 ALA and 12·6% for placebo patients, respectively (Table 6).

**Table 6:** Recurrent lesions at 6-month (FUP1) and 12-month (FUP2) follow-up

	BF-200 ALA (ALA-AK-CT003)			BF-200 ALA (ALA-AK-CT002)^a^			MAL (ALA-AK-CT002)^a^		
			
	Recurrent^b^, %	*P*_i_	*P*_i_ * CR_L_	Recurrent^b^, %	*P*_i_	*P*_i_ * CR_L_	Recurrent^b^, %	*P*_i_	*P*_i_ * CR_L_
*Overall*									
FUP1	7·4	0·928	0·752	7·0	0·931	0·733	6·6	0·935	0·589
FUP2^c^	16·7	0·843	0·684	21·7	0·855	0·525	25·4	0·813	0·401
*Light spectrum*									
FUP1									
Narrow	1·8	0·982	0·945	4·1	0·960	0·818	5·2	0·949	0·622
Broad	12·7	0·878	0·620	11·2	0·890	0·633	8·4	0·916	0·554
FUP2^c^									
Narrow	7·0	0·932	0·897	20·6	0·839	0·591	24·1	0·813	0·435
Broad	26·4	0·760	0·536	23·0	0·883	0·445	27·2	0·812	0·363

AK, actinic keratosis; ALA, 5-aminolaevulinic acid; MAL, methyl aminolaevulinate; *P*_i_, probability that a completely cleared lesion remains completely clear up to FUP1 or FUP2; *P*_i_ * CR_L_, probability that a lesion is fully cleared during photodynamic therapy and remains totally clear until the follow-up visit (product of *P*_i_ and efficacy rate at the end of the clinical trial).

a Only lesions of complete responders are considered;

b the percentage is calculated according to the number of lesions at the respective visit;

c recurrences are cumulated.

The probabilities of lesions remaining totally clear up to FUP1 were very similar for BF-200 ALA- and MAL-treated patients with values around 0·93. Again, there were slight advantages for BF-200 ALA at FUP2 with a *P*_2_ of 0·855 for BF-200 ALA-treated patients in study ALA-AK-CT002 vs. 0·813 for MAL-treated patients. In the ALA-AK-CT003 study the corresponding probability was 0·843 (Table 6).

The differences in the lesion recurrence rates between the two pivotal studies are likely to be caused by the different way of counting recurrent lesions. While all lesions were recorded individually in study ALA-AK-CT003, all lesions of a patient were counted as recurrent in study ALA-AK-CT002 if this patient received any AK therapy in between the scheduled visits or if the number of recurrent lesions was not recorded by the investigator. After 12 months (FUP2 of ALA-AK-CT002), 78 out of 127 recurrent lesions in the BF-200 ALA group, 104 out of 140 recurrent lesions in the MAL group and eight out of eight lesions in the placebo group belonged to the aforementioned patients. These high proportions illustrate that the procedure leads to an overestimation of lesion recurrence in the ALA-AK-CT002 study. It should not, however, affect any differences between lesion recurrence in the BF-200 ALA and MAL groups.

The probability of reaching total lesion clearance 12 months after the last PDT (*P*_2_ * CR_L_) for BF-200 ALA was 0·525 in ALA-AK-CT002 and 0·684 in ALA-AK-CT003. For MAL the probability was 0·401, considerably lower than the probabilities with BF-200 ALA (Table 6).

#### Subgroups

In both pivotal studies a difference in the number of subjects with recurrent AK lesions treated either with narrow- or broad-spectrum light sources was obtained at both FUP visits for all verum groups (Table 7).

**Table 7:** Patient subgroups completely cleared at 6-month (FUP1) and 12-month (FUP2) follow-up

	BF-200 ALA (ALA-AK-CT003)			BF-200 ALA (ALA-AK-CT002)			MAL (ALA-AK-CT002)		
			
Subgroup/visit	Patients still completely cleared^a^, %	*P*_i_	*P*_i_ * CR_P_	Patients still completely cleared^a^, %	*P*_i_	*P*_i_ * CR_P_	Patients still completely cleared^a^, %	*P*_i_	*P*_i_ * CR_P_
*Light spectrum*									
Broad									
FUP1	75·0	0·760	0·403	74·7	0·750	0·545	76·7	0·767	0·483
FUP2	60·9	0·630	0·334	56·3	0·567	0·412	50·7	0·507	0·319
Narrow									
FUP1	88·9	0·889	0·774	86·1	0·864	0·749	86·4	0·867	0·592
FUP2	76·9	0·775	0·675	60·2	0·613	0·531	59·3	0·597	0·408
*AK severity at baseline*									
Grade I									
FUP1	100	1·000	0·789	78·1	0·788	0·667	86·5	0·865	0·821
FUP2	80·0	0·800	0·632	69·7	0·697	0·590	64·9	0·649	0·615
Grade II									
FUP1	75·0	0·757	0·471	81·4	0·816	0·643	80·3	0·807	0·482
FUP2	64·7	0·669	0·417	55·9	0·569	0·448	52·1	0·525	0·313
*Target area*									
A: face/forehead									
FUP1	79·3	0·800	0·564	82·9	0·832	0·693	83·7	0·840	0·651
FUP2	71·4	0·729	0·514	59·6	0·607	0·506	60·6	0·609	0·472
B: bald scalp									
FUP1	92·3	0·923	0·545	76·6	0·766	0·554	82·6	0·826	0·352
FUP2	84·6	0·846	0·500	57·4	0·574	0·415	52·2	0·522	0·222
A + B									
FUP1	77·8	0·778	0·500	79·2	0·800	0·625	74·1	0·741	0·417
FUP2	37·5	0·419	0·269	54·2	0·554	0·433	37·0	0·370	0·208
*Complete responder: after 1 PDT*									
FUP1	89·2	0·893	0·424	86·4	0·864	0·423	83·1	0·832	0·312
FUP2	71·4	0·726	0·345	65·5	0·659	0·323	59·6	0·596	0·224

AK, actinic keratosis; ALA, 5-aminolaevulinic acid; MAL, methyl aminolaevulinate; PDT, photodynamic therapy; *P*_i,_ probability that a completely cleared patient remains completely clear up to FUP1 or FUP2; *P*_i_ * CR_P,_ probability that a patient is fully cleared from all lesions during PDT and remains totally clear until the follow-up visit (product of *P*_i_ and efficacy rate at the end of the clinical trial).

a The percentage is calculated according to the number of patients at the respective visit.

In the comparative study ALA-AK-CT002, a similar number of patients was still completely clear 12 months after the last treatment with BF-200 ALA or MAL and illumination with LED devices (60·2% with BF-200 ALA and 59·3% with MAL). With broad-spectrum devices this clearance rate was, with 56·3%, slightly lower for BF-200 ALA, while MAL showed a clearly reduced rate of 50·7% (Table 7). In study ALA-AK-CT003, a strong difference was observed between narrow and broad light spectrum lamps, with 76·9% and 60·9% of the BF-200 ALA patients remaining completely clear, respectively (Table 7).

At FUP2 after 12 months, the probabilities of lesions remaining totally cleared after treatment with LED lamps were 0·932 and 0·839 for BF-200 ALA (studies ALA-AK-CT003 and ALA-AK-CT002, respectively) and 0·813 for MAL (Table 6).

The analysis of the various subgroups shown in Table 7 showed that the recurrence rates after BF-200 ALA treatment compared with MAL treatment were in no cases dramatically different. However, after 12 months’ FUP slightly lower recurrences were observed in all analysed subgroups of BF-200 ALA-treated patients, leading to the conclusion that there was a general tendency to slightly lower recurrence rates with this product (Table 7).

Mostly due to the significantly higher efficacy of BF-200 ALA, the probability of a patient being totally clear of all lesions after 12 months of FUP (*P*_2_ * CR_P_) was to the advantage of BF-200 ALA compared with MAL in all subgroups analysed (Table 7).

### Cosmetic outcome

The cosmetic outcome was assessed by the investigator at the 6-month FUP as very good or good in 39·7% and 43·1% subjects in the BF-200 ALA groups (study ALA-AK-CT002 and ALA-AK-CT003, respectively), in 42·6% of subjects in the MAL group (study ALA-AK-CT002), and 34·8% and 44·1% of subjects in the placebo groups (studies ALA-AK-CT002 and ALA-AK-CT003, respectively). An unsatisfactory or impaired assessment was obtained in 14·3% and 7·0% of subjects treated with BF-200 ALA (ALA-AK-CT002 and ALA-AK-CT003, respectively), and in 9·0% of MAL-treated patients. Higher values were obtained for placebo with 13·7% and 20·6% in the respective studies.

At the 12-month FUP, the cosmetic outcome was judged as very good or good in 38·9% and 45·0% of subjects in the BF-200 ALA groups (ALA-AK-CT002 and ALA-AK-CT003, respectively), in 41·1% of subjects in the MAL group, and in 32·8% and 46·9% of subjects in the placebo groups (ALA-AK-CT002 and ALA-AK-CT003, respectively). Unsatisfactory or impaired outcome was reported to a similar extent in all groups (15·0% and 14·1% of BF-200 ALA-treated patients, 16·5% of MAL-treated subjects, 15·7% and 18·8% of placebo patients, respectively).

### Safety and tolerability

Sixty-one AEs were reported during the FUP of study ALA-AK-CT002, which occurred to a similar extent in the three study arms (11·6%, 11·3% and 8·8% in the BF-200 ALA, the MAL and the placebo groups, respectively). All events were classified as unrelated with the exception of one subject with SCC in the BF-200 ALA group, two patients with basal cell carcinomas (BCC) in the MAL group and one subject each with Bowen’s disease in the MAL and placebo groups. No AEs were assessed in the ALA-AK-CT003 study.

During the ALA-AK-CT002 FUP study, 10 SAEs were reported in eight subjects (three for subjects treated with BF-200 ALA, five for subjects who received MAL). Four SAEs were fatal. The SAEs occurred between about 6 weeks after the last PDT and about 1 year after the last PDT and the investigators considered them unrelated or unlikely to be related to the PDT. No SAEs were noted during the ALA-AK-CT003 FUP.

New lesions occurred in all patient groups of the pivotal studies, affecting from 41·7% to 56·1% of the patients after 12 months, with the exception of the ALA-AK-CT003 placebo arm, where for unknown reasons the percentage was only 20·6% (Table 8). In the ALA-AK-CT002 study, the percentage of patients developing new AK lesions was higher for noncomplete responders than for complete responders (65% vs. 36%, 64% vs. 43% and 65% vs. 31% for BF-200 ALA-, MAL- and placebo-treated patients, respectively). Study ALA-AK-CT003 showed similar numbers in the BF-200 ALA group with 39·6% for complete responders vs. 47·4% for noncomplete responders, but clearly different figures in the placebo group (24·1% vs. 0%).

**Table 8:** New lesions and skin cancer in the target areas

	ALA-AK-CT003		ALA-AK-CT002		
		
	BF-200 ALA, *n* (%)	Placebo, *n* (%)	BF-200 ALA, *n* (%)	MAL, *n* (%)	Placebo, *n* (%)
New AK					
FUP1 (6 months)	16/72 (22·2)	4/34 (11·8)	51/237 (21·5)	56/236 (23·7)	18/66 (27·3)
FUP2^a^ (12 months)	14/71 (19·7)	3/32 (9·4)	48/234 (20·5)	59/231 (25·5)	19/64 (29·7)
Overall	30/72 (41·7)	7/34 (20·6)	99/237 (41·8)	115 (48·7)	37/66 (56·1)
Nonmelanoma skin cancer					
FUP1 (6 months)	2/72 (2·8)	1/34 (2·9)	3/237 (1·3)	2/236 (0·8)	3/66 (4·5)
FUP2^a^ (12 months)	1/71 (1·4)	0	5/234 (2·1)	6/231 (2·6)	1/64 (1·6)
Overall	3/72 (4·2)	1/34 (2·9)	8/237 (3·4)	8/236 (3·5)	4/66 (6·3)

Numbers indicate the affected patients/all patients assessed at the particular follow-up (FUP) visit. AK, actinic keratosis; ALA, 5-aminolaevulinic acid; MAL, methyl aminolaevulinate.

a Data for FUP visit 2 reflect the number of patients who developed new lesions or nonmelanoma skin cancer since FUP visit 1.

Nonmelanoma skin cancers (SCC, BCC) developed in the treatment area in only a few patients at a very similar extent in most study groups, with the exception of a slight increase in the ALA-AK-CT002 placebo group (Table 8). No melanomas were reported in either study. Interestingly, most of the patients with nonmelanoma skin cancer were noncomplete responders (14/20 in ALA-AK-CT002, two of four in ALA-AK-CT003) or had a history of skin diseases including AK for several years (18/20 in ALA-AK-CT002, four of four in ALA-AK-CT003).

## Discussion

BF-200 ALA is a new nanoscale oil-in-water emulsion of ALA for PDT, which was recently shown to be a very effective and safe treatment option for AK lesions.^6^,^7^ PDT is recommended as a first-line therapy for the treatment of AK due to its high efficacy, the possibility of treating extended skin areas and its superior cosmetic outcome compared with other treatment modalities.^1^ However, long-term follow-up data of controlled clinical trials are limited and only a few publications have reported recurrence rates after ALA or MAL PDT treatment over a longer follow-up period.^9^,^11^

The present article summarizes 6- and 12-month FUP data collected in two pivotal phase III studies.^6^,^7^ The reported studies compared the clearance and recurrence rates, new lesion formation and cosmetic outcome in patients with AK treated with BF-200 ALA or placebo, and one of the studies compared the results for BF-200 ALA with those for a commercially available MAL formulation. The data confirm that PDT is a highly effective and safe therapy for AK. Both PDT drugs displayed low recurrence rates, in favour of BF-200 ALA over MAL. The probability that complete responders remained clear 12 months after the last treatment was 0·59 or 0·71 for BF-200 ALA (study ALA-AK-CT002 and ALA-AK-CT003, respectively) and 0·56 for MAL (study ALA-AK-CT002). Although these recurrence rates with BF-200 ALA are not substantially lower than with MAL, the difference is reflected in all subgroups analysed, illustrating a general tendency to slightly lower recurrence rates.

Nevertheless, the probability of clearance 12 months after PDT is strongly in favour of BF-200 ALA due to its statistically significantly higher efficacy. These probabilities, denoted *P*_2_ * CR_P_, were 0·47 for BF-200 ALA vs. 0·36 with MAL for all lamps, and 0·53 vs. 0·41 for narrow-spectrum lamps, respectively, in study ALA-AK-CT002. Thus, calculating a patient’s long-term prognosis as the combination of efficacy and recurrence rates demonstrates the strong superiority of BF-200 ALA compared with MAL. It is worth noting that *P*_2_ * CR_P_ values in patients who were completely cleared after a single PDT, were still better 12 months after BF-200 ALA treatment than 6 months after MAL treatment (Table 7).

The two pivotal trials documented a strong influence of the applied lamp sources on PDT efficacy. Narrow-wavelength lamps generated much better clinical efficacy than broad-wavelength lamps.^6^,^7^ Therefore, it was of particular interest to compare the recurrence rates of these subgroups. For most subgroups, patients illuminated with narrowband LED lamps displayed lower recurrence rates than those illuminated with broadband lamps. Therefore, the advantage of higher efficacy provided by the LED lamps is at least maintained or even enhanced during the 12 months following PDT.

Studies describing recurrence rates of conventional therapies are rare and mostly poorly controlled. The recurrence rates described^13^ after 1 year were similar or worse than those described here. A better data basis is available for PDT drugs, where several authors published FUP results of phase III studies of AK PDT. The life-table analysis, in which the probability *P*_2_ * CR_P_ expresses the likelihood that a patient is completely cleared during treatment and remains clear of all lesions for 12 months after treatment, should serve as the basis to choose the optimal therapy for the patients. Our data illustrate that 53% (study ALA-AK-CT002) to 68% (study ALA-AK-CT003) of patients treated with BF-200 ALA and LED lamps, and 41% of patients treated with MAL and LED lamps are cleared of all lesions during treatment and remain free of all lesions for at least 12 months after treatment. This treatment success is clearly above the values achieved in controlled trials with Levulan Kerastick® (DUSA, Wilmington, MA, U.S.A.) (40%),^9^ ALA patch (21–45%),^10^,^11^ cryotherapy (29%)^10^,^11^ or of a recently approved ingenol mebutate (0·015%)-containing gel (20%)^12^ (Table 9). In the studies cited here Levulan Kerastick was, according to its product specification, used in combination with blue light, the ALA patch also with LED lamps.

**Table 9:** Results of long-term follow-up studies using LED lamps^6^,^7^,^9^–^12^

	ALA^a^,^6^,^7^ (BF-200 gel, 78 mg g^−1^)	MAL^a^,^7^ (cream, 160 mg g^−1^)	ALA^a^,^10^,^11^ (patch, 2 mg cm^–^^2^)	ALA^b^,^9^ (2-compound system, 156 mg g^−1^)	Cryotherapy^10^,^11^	Ingenol mebutate^12^ (gel, 150 μg g^−1^)
Completely cleared patients at the end of clinical study, %	CT002: 85^c^; CT003: 96^d^	CT002: 68^c^	AK3: 62^c^; AK4: 67^d^	60^d^,^e^	52^c^	42^g^
Estimated patient-wise AK clearance rate related to number of patients pretreatment 12 months after last treatment, completely cleared patients, %	CT002: 53^a^; CT003: 68^a^	CT002: 41^a^	AK3: 21^a^; AK4: 45^a^	40^f^	29	20^h^

Concentrations refer to free acid. AK, actinic keratosis; ALA, 5-aminolaevulinic acid; MAL, methyl aminolaevulinate.

a Treated with LED lamps;

b using BlueU (417 nm) lamp;

c full analysis set/intent to treat;

d per-protocol population;

e 3–5 months after last treatment;

f 10–12 months after last treatment;

g 57 days after last application;

h 57 days + 12 months.

The lesion recurrence rates observed in the 12-month FUP assessments for lesions treated with BF-200 ALA and LEDs are in a similar range (21% in study ALA-AK-CT002) or clearly below (7% in study ALA-AK-CT003) the values provided in the published literature.^9^,^11^,^14^ On average with all lamps the recurrence rates were 22% in study ALA-AK-CT002 and 17% in study ALA-AK-CT003. Lesion recurrence rates for MAL were slightly higher, with 24% for LED lamps and 25% on average for all lamps. However, it must be taken into consideration that lesion recurrence in study ALA-AK-CT002 is overestimated as, for subjects who received AK therapy between visits or for whom the number of recurrent lesions was not documented, all lesions were classified as recurrent, irrespective of the actual recurrence. This aspect is most relevant in the recurrence evaluation at FUP2 in which 61% of recurrent lesions in the BF-200 ALA and 74% of recurrent lesions in the MAL group belonged to the aforementioned patient group. All the general tendencies observed for total patient recurrence were paralleled and confirmed by the data obtained when the occurrence of individual lesions was calculated. Here also, recurrence rates with MAL were slightly higher than those for BF-200 ALA in all subgroups.

The assessment of the cosmetic outcome as very good or good for BF-200 ALA- and MAL-treated patients differed only slightly from the evaluation at the end-of-study visit (BF-200 ALA: 47·6% and 43·1% at the end of study vs. 45·0% and 38·9% at FUP2, for ALA-AK-CT003 and ALA-AK-CT002, respectively; MAL: 45·2% vs. 41·1%, respectively). Placebo values increased from 25% to 46·9% in study ALA-AK-CT003 and changed from 36·4% to 32·8% in the ALA-AK-CT002 study.^6^,^7^ The lower values in the placebo groups might be influenced by the higher percentage of additional AK therapies applied subsequent to the studies. Application of conventional AK therapies to recurrent patients might also explain the increase of unsatisfactory or impaired values in the verum groups during FUP.

In conclusion, treatment with BF-200 ALA revealed a high efficacy in the treatment of AKs which is maintained over a 1-year FUP. Total patient clearance rates are significantly higher with BF-200 ALA than with MAL, and this advantage may even be enhanced by the tendency to lower recurrence rates.

**What’s already known about this topic?**

- BF-200 ALA is a stable nanoemulsion-based gel formulation of 5-aminolaevulinic acid (ALA) for photodynamic therapy (PDT) of actinic keratosis (AK), which demonstrated significantly higher efficacy compared with a registered methyl aminolaevulinate (MAL) cream.

**What does this study add?**

- This study gives 6- and 12-month follow-up results of two pivotal phase III studies with BF-200 ALA for PDT of AK in comparison to placebo and a registered MAL cream.
- It provides a comparison of recurrence rates after use of different light sources for PDT of AK.
